# Dissociable Electroencephalograph Correlates of Visual Awareness and Feature-Based Attention

**DOI:** 10.3389/fnins.2017.00633

**Published:** 2017-11-13

**Authors:** Yifan Chen, Xiaochun Wang, Yanglan Yu, Ying Liu

**Affiliations:** ^1^School of Kinesiology, Shanghai University of Sport, Shanghai, China; ^2^Key Laboratory of Exercise and Health Sciences of Ministry of Education, Shanghai University of Sport, Shanghai, China

**Keywords:** exogenous attention, visual consciousness, event related potentials (ERP), reaction time, cueing paradigm

## Abstract

**Background:** The relationship between awareness and attention is complex and controversial. A growing body of literature has shown that the neural bases of consciousness and endogenous attention (voluntary attention) are independent. The important role of exogenous attention (reflexive attention) on conscious experience has been noted in several studies. However, exogenous attention can also modulate subliminal processing, suggesting independence between the two processes. The question of whether visual awareness and exogenous attention rely on independent mechanisms under certain circumstances remains unanswered.

**Methods:** In the current study, electroencephalograph recordings were conducted using 64 channels from 16 subjects while subjects attempted to detect faint speed changes of colored rotating dots. Awareness and attention were manipulated throughout trials in order to test whether exogenous attention and visual awareness rely on independent mechanisms.

**Results:** Neural activity related to consciousness was recorded in the following cue-locked time-windows (event related potential, cluster- based permutation test): 0–50, 150–200, and 750–800 ms. With a more liberal threshold, the inferior occipital lobe was found to be the source of awareness-related activity in the 0–50 ms range. In the later 150–200 ms range, activity in the fusiform and post-central gyrus was related to awareness. Awareness-related activation in the later 750–800 ms range was more widely distributed. This awareness-related activation pattern was quite different from that of attention. Attention-related neural activity was emphasized in the 750–800 ms time window and the main source of attention-related activity was localized to the right angular gyrus. These results suggest that exogenous attention and visual consciousness correspond to different and relatively independent neural mechanisms and are distinct processes under certain conditions.

## Introduction

Theories of consciousness posit that neural amplification plays a key role in information successfully gaining access to awareness (Dennett, 1991; Baars, 1997; Dehaene et al., 1998; Edelman, 2003). Attention selectively enhances neural responses to relevant targets (Corbetta et al., 1990; Reynolds and Chelazzi, 2004; Müller et al., 2006) and therefore, sensory amplification via attention fosters visual awareness (Dehaene et al., 2006). Interestingly, this intuitively appealing theory has been challenged by several empirical findings. While attention and awareness are related, they have distinct and independent neural processes (Koivisto et al., 2006; Wyart and Tallon-Baudry, 2008; Davoodi et al., 2015), and these findings are difficult to reconcile with the theory that attention acts as a gateway to awareness.

There are experimental techniques that help separate the intertwined concepts of spatial attention and visual awareness. For example, Wyart and Tallon-Baudry recorded magnetoencephalographic signals while human subjects attended toward or away from faint stimuli that were reported as consciously seen in only half of the trials (Wyart and Tallon-Baudry, 2008). Visually identical stimuli could thus be attended or not attended and consciously seen or not seen. In their study, participant attention was directed to different locations and they found a double disassociation between spatial attention and visual awareness. We were thus inspired to test if a similar disassociation occurs for feature-based attention and awareness.

Different forms of attention may interact uniquely with awareness. Behaviorally, exogenous attention (reflexive attention) does not necessarily act as a gateway to gain access to awareness. There is growing evidence that endogenous attention (voluntary attention) (James, 1890), can modulate subliminal processing (e.g., Kanai et al., 2006; Kentridge et al., 2008; Webb et al., 2016) and for reviews, see (van Boxtel et al., 2010; Tsuchiya and Koch, 2016) and while exogenous attention also modulates subliminal processing (Sumner et al., 2006; Schmidt and Schmidt, 2010; Norman et al., 2015), it does so in a unique manner from endogenous attention (Hsu et al., 2011). However, at the neural level, it has been suggested that the coupling of attention and consciousness is stronger during exogenous attention than endogenous attention. While endogenous attention is electrophysiologically dissociated from conscious perception (Koivisto et al., 2006; Davoodi et al., 2015), exogenous attention is not (Chica et al., 2010, 2012), consistent with the hypothesis that exogenous attention is an important antecedent of conscious experience. Furthermore, endogenous attention modulates conscious perception, but only when phasic alerting or bottom-up activation is increased (Botta et al., 2014), demonstrating the necessity of exogenous attention in conscious experience. In accordance, exogenous attention may have a more important role in accessing awareness than endogenous attention. Considering the inconsistencies between the behavioral and neural findings regarding exogenous attention, the question of whether exogenous attention and visual awareness rely on independent mechanisms under certain circumstances remains unanswered.

The findings presented above which support dissociation of neural activity between spatial attention and visual consciousness have mostly involved event related potentials (ERP). ERP is often used for its excellent time resolution. Koivisto et al. observed an awareness-related posterior negative amplitude shift in the 130–320 ms period after stimulus presentation, independent of the scope of attention (global vs. local) (Koivisto et al., 2006). They also found awareness-related ERPs at approximately 400 ms, peaking at parietal sites, but the awareness-related effect was attenuated in the local attention condition, suggesting a tight interaction between attention and consciousness. Davoodi et al. (2015) also reported that parieto-occipital areas are the most relevant areas for dissociation between attention and consciousness. Some of the ERP components (P100, N150, and P300) changed with attention or consciousness (Davoodi et al., 2015).

**Figure 1:**
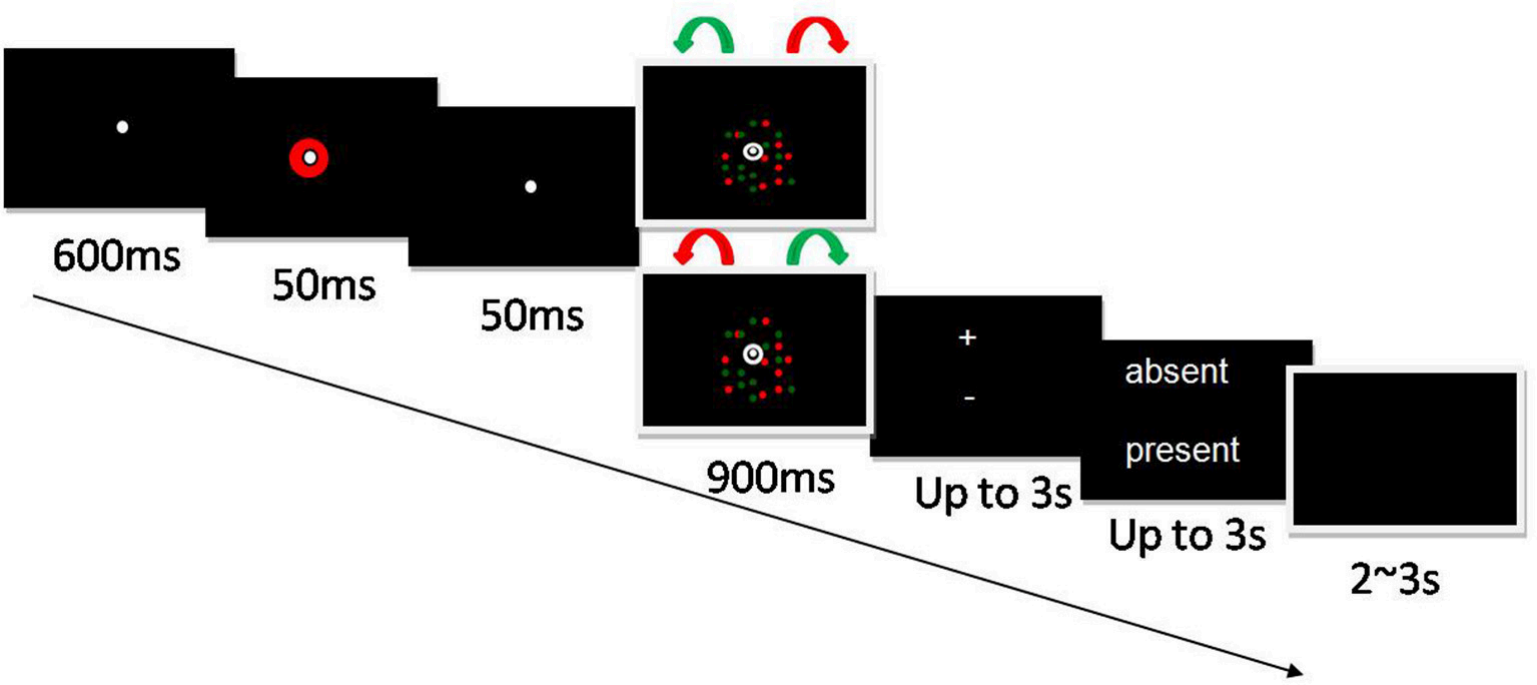
Experimental design. Two populations of dots, one red and one green, were shown on each trial. Each population was composed of 50 dots, and all targets were in an annulus 1–3 dva from fixation. One colored population rotated clockwise and the other counterclockwise. Which color rotated in which direction was randomly assigned. Following a 600 ms fixation screen, a 50 ms colored cue, and then another 50 ms fixation screen, the colored dots were shown rotating for 900 ms. On 87% of trials, one of the two dot populations either sped up or slowed down at an acceleration/deceleration rate determined by a prior one-up-one-down staircase procedure that led to a 50% detection rate for attended colors for each participant (see below). The color of the population that changed speed was congruent with the color of the cue in only half of the trials. This seemingly “meaningless” cue is used to manipulate rapid and reflective exogenous attention. On the remaining 13% of trials, the dots rotated at their original, uniform velocity (6 dva/s). Subjects were asked to answer two successive questions: first, whether there was an acceleration or deceleration of the target stimuli (two-alternative forced choice), and second, whether they thought one dot population moved at a variable speed.

Therefore, electroencephalograph (EEG) recordings were conducted while subjects attempted to detect faint speed changes of colored rotating dots. The speed change was perceived consciously in approximately half of the trials. In addition, in half of the trials the color of the rotating dots was attended, in that the participant was exposed to a target cue of the same color (Figure 1). In this feature-based exogenous attention by visual awareness factorial design, each stimulus could be classified as attended (color of the cue was congruent with color of the rotating dots that underwent a speed change) or unattended (color of the cues was incongruent with the color of the rotating dots that underwent a speed change). An uninformative colored cue was flashed at the beginning of each trial that sometimes matched the color of the dot population that changed in speed. This method has previously been proposed as an exogenous cuing mechanism for feature-based attention (Lin et al., 2011). In addition, trials were either aware (subject perceived the speed change) or unaware (speed change was not perceived). We took advantage of this factorial design and EEG data to identify the neural correlates of color-based exogenous attention, of awareness, and of the interaction between color-based exogenous attention and awareness.

## Methods

### Subjects

Seventeen volunteers with normal or corrected-to-normal vision participated in the study. Data from one male participant were discarded due to a high false alarm rate and therefore, data from sixteen participants (all right handed; 8 women and 8 men; ranging in age from 18 to 26 years, mean ± SEM = 21.75 ± 2.32) were entered into analysis. All participants had normal color vision as assessed by the Ishihara Color Vision Test. Participants signed written informed consent and were paid for their participation. All study procedures adhered to the ethical guidelines of the Declaration of Helsinki and were approved by the local ethics committee (Ethics Committee, Shanghai University of Sport, Shanghai, China).

### Stimuli

Stimuli were presented on a calibrated computer screen (resolution of 1,024 × 768 pixels and refresh rate of 60 Hz) positioned at 1.1 m from the eyes of the participant. The effective luminance of the stimulus was calibrated with a Sanpometer SM208 Luminance Meter (Sanpo Instrument Co., Ltd, Shenzhen, China). Stimulus presentation was controlled by the Psychtoolbox package for Matlab (Brainard, 1997; Pelli, 1997). All displays had a black background. The fixation mark at the center of the screen was a white disk with a degree of visual angle (dva) of 0.15 degrees. The cue was a colored circle (either red or green) with a radius of 1 dva. The luminance of the red circle was fixed throughout the experiment at 1.57 cd/m2 and the luminance of the green circle was set individually for each subject at a perceptual equiluminance with red using a heterochromatic flicker method (see below).

The target consisted of two populations of dots, one red and one green. Each population was composed of 50 dots, and all targets were in an annulus 1–3 dva from fixation. One colored population rotated clockwise and the other counterclockwise. Which color rotated in which direction was randomly assigned.

### Task and procedure

Following a 600 ms fixation screen, a 50 ms colored cue, and then another 50 ms fixation screen, the colored dots were shown rotating for 900 ms. On 87% of trials, one of the two dot populations either sped up or slowed down at an acceleration/deceleration rate determined by a prior one-up-one-down staircase procedure that led to a 50% detection rate for attended colors for each participant (see below). The color of the population that changed speed was congruent with the color of the cue in only half of the trials. This seemingly “meaningless” cue is used to manipulate rapid and reflective exogenous attention. On the remaining 13% of trials, the dots rotated at their original, uniform velocity (6 dva/s). Participants were then presented successively with two response screens for a maximum of 3 s. The first screen was a two-alternative forced-choice speed-change discrimination: subjects were asked to determine whether there was an acceleration or deceleration of the target stimuli. Subjects were then asked whether they thought one dot populations moved with at a variable speed, by answering “present” or “absent.” In both tasks, the two choices were presented above and below the fixation point. Subjects pressed the upper or lower response key with their right index or middle finger. The upper or lower position of the responses in the response screens was randomized from trial to trial to avoid systematic stimulus-response mapping and prevent motor preparation during target presentation. The location of the response options did not overlap the annulus to prevent any masking effect. Participants were instructed to answer quickly and accurately and were explicitly told to make a choice even if they were not sure of their response. During the inter-stimulus interval (2–3 s), a black background was displayed.

To minimize perceptual differences between red and green, the perceptual equiluminance of the two colors was determined for each subject individually using a heterochromatic flicker method as follows. The color of the two dot populations (rotated around the fixation/target point) alternated between red and green at 30 Hz against a black background. Participants were instructed to minimize the sensation of flickering by clicking on two buttons that increased or decreased the luminance of the green (Wagner and Boynton, 1972). Subjects underwent eight trials; two each of red or green clockwise-rotating dots on a bright or dark starting point, presented in a random order. The average luminance of green at minimal flicker was used in the following sessions. Establishing equiluminance required approximately 5 min.

Once the two colors were set at perceptual equiluminance, the acceleration or deceleration at which subjects could detect 50% of speed-change was determined using a “one-up-one-down” staircase procedure. Trials were identical to those described above, except that the acceleration or deceleration was varied from trial to trial depending on whether the speed-change was perceived in the previous trial of the same type. Four independent staircases (one for each condition: attended/unattended × acceleration/deceleration) were intermixed during the session. However, due to time limitation, only the staircases for the attended condition of each color were run until convergence. The thresholds used in the following sessions were those obtained for attended targets. This portion of the procedure required approximately 20 min.

The staircase session was followed by eight recording sessions for data collection (mean duration, 8 min/session), during which continuous EEG signals were recorded. Each session consisted of 92 trials (80 speed-change-present trials, including 40 of the attended color and 40 of the unattended color; and 12 speed-change-absent trials; six of the attended color and six of the unattended color).

### EEG data acquisition

Continuous EEG signals were collected using the Brain Vision Recorder 2.0 system (Brain Products Company, Germany) with an Easy-Cap containing 64 electrodes placed according to the International 10–20 system. EEG recordings were referenced against the FCz site, with AFz as the ground electrode. Vertical and horizontal electrooculogram (EOG) signals were also collected for offline eye movement rejection. The continuous EEG signal was amplified with a band-pass of 0.01–100 Hz and then digitized at a sampling rate of 1,000 Hz using a BrainAmp amplifier. The impedance was <5 kΩ.

### EEG data analysis

Offline data from the EEG recordings were analyzed using Vision Analyzer 2.0 system (Brain Products Company, Germany). Prior to further data analyses, EOG artifacts were reduced using the build-in method (Gratton et al., 1983; Miller et al., 1988). EEG recordings were then segmented into trials from 600 ms before cue onset to 4,000 ms after target onset. Trials with obvious movement or muscle artifacts were discarded. Trials were rejected if the voltage exceeded ±80 μV.

The remaining speed-change-present trials were averaged for each subject and each of the following four conditions: aware-attended, unaware-attended, aware-unattended and unaware-unattended. During “attended” trials, the color of the speed-change dot population was the same as that of the cue while in “unattended” trials the color of the speed-change dot population was different from the cue. Absent reported trials were referred to as “unaware” trials while “aware” trials were reported as present and the speed-change dot population was correctly identified. The mean number of available trials per subject was 165 (range 106–243, *SD* = 39), 144(range 67–199, *SD* = 45), 161 (range 82–243, *SD* = 45) and 147 (range 68–216, *SD* = 50). Averaged data were low-pass filtered at 30 Hz, and baseline corrected using the 400 ms preceding cue onset.

Taking into account that the neural bases of consciousness and attention are disputed and in addition, that there is no prior ERP literature using the same paradigm as ours, we chose a cluster-permutation test to explore the ERPs. The cluster-permutation method tests differences without predefinition of time or area, but with a strict control for multiple comparisons (i.e., sensors and time samples). This method is therefore only sensitive to activity lasting over a long duration and a large area. Therefore, the specific time ranges of interest were determined a priori (i.e., independently from feature-based attention and perceptual awareness) to raise the sensitivity of the analysis. The periods of interest were predefined around peaks where brain activity was robust and differences would be more likely observed.

**Figure 2:**
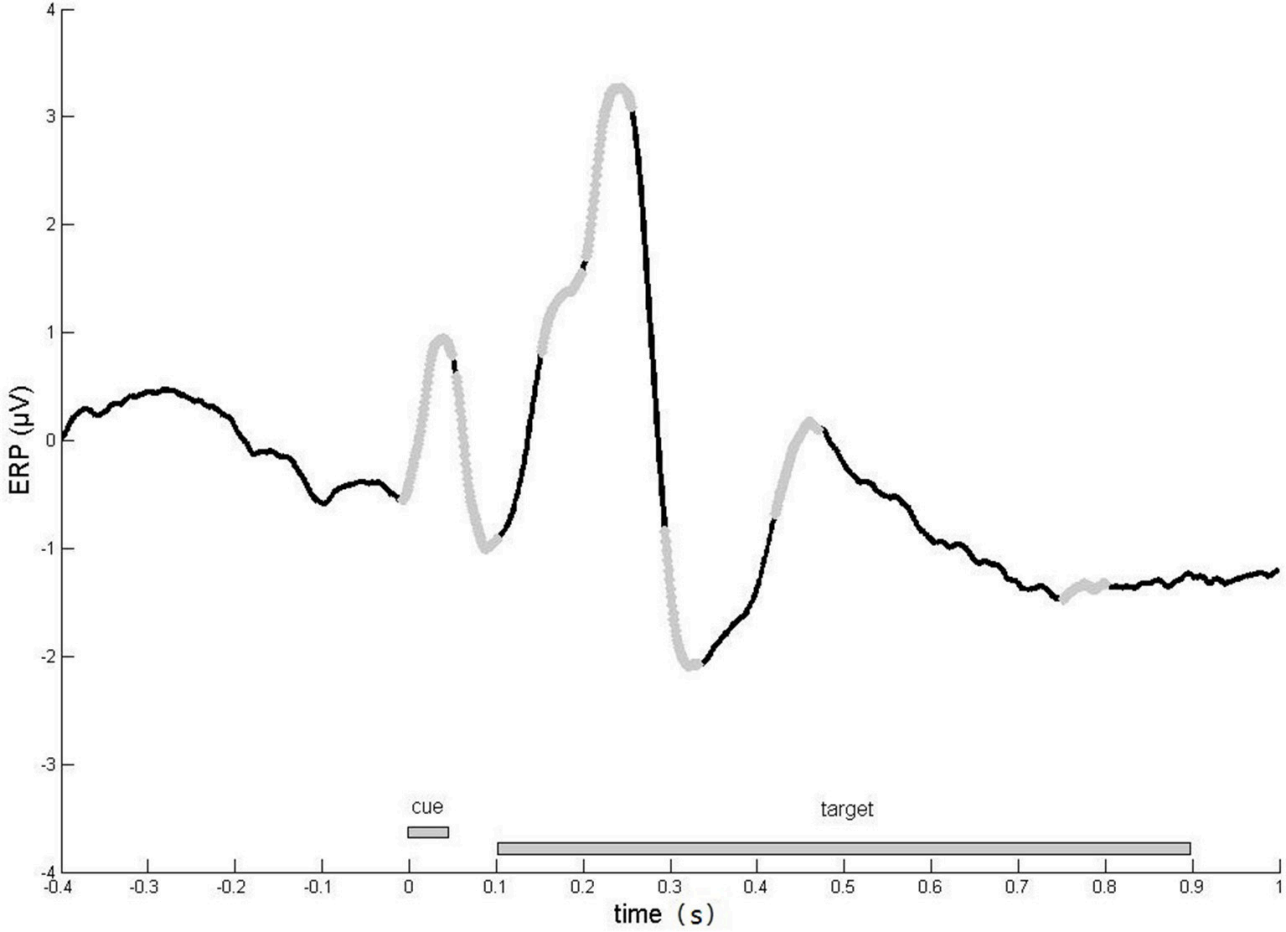
Time course of evoked responses. Averaged evoked responses across all ERP sensors, experimental conditions and subjects. We focused our analyses on the time windows (light gray line), in which robust responses were observed.

To define periods of interest, averages of event-related potentials (ERPs) were computed for each subject, electrode, and experimental condition. Based on grand average curves (Figure 2), seven time windows were defined: 0–50, 50–100, 150–200, 200–250, 280–330, 420–470, and 750–800 ms.

A repeated measures, two-tailed cluster-based permutation test proposed by Maris and Oostenveld (2007) (implemented in the Fieldtrip Toolbox; Oostenveld et al., 2011) was used to determine the neural correlates of color-based attention of awareness while controlling for multiple comparisons (sensors, time samples). In detail, repeated measures *t*-tests were run for awareness (aware/unaware) and attention (attended/unattended) for each signal sample (one sensor, one time point) of the evoked response to speed-change-present trials. For each main effect, (one-tailed *p* ≤ 0.1) samples were clustered based on time and space adjacency. Two sensors were considered neighbors if they were separated by <4 cm. For a two-sided test, the clustering was performed separately for samples with a positive and a negative *t*-value. Each cluster defined in space and time by this procedure was then assigned a cluster-based value equal to the sum of the *t*-values of all the samples belonging to the cluster. To test whether this cluster-level statistic was obtained by chance, the condition labels of the original event-related field data of each subject were randomly shuffled. The clustering procedure was then applied to the randomized data, and the maximal cluster *t*-value was measured for each factor in each period of interest. By repeating the random assignment of condition labels to EEG data 1,000 times, the distribution of the maximum cluster level *t*-value under the null hypothesis could be estimated separately for each main effect in each period of interest. If the original statistic was greater (positive cluster level *t*-value) or smaller (negative cluster level *t*-value) than 97.5% of the values obtained on randomized data, then the null hypothesis was rejected with a total *p* < 0.05. The advantage of this method is that the multiple comparisons are intrinsically controlled by using the maximum statistics.

### Source localization

Cortical current density mapping was obtained using a distributed model consisting of 15,000 current dipoles. Dipole locations and orientations were loosely mapped to the cortical mantle of a generic brain model built from the standard brain of the Montreal Neurological Institute using BrainVISA software (http://brainvisa.info). Source localization and surface visualization were performed with BrainStorm (Tadel et al., 2011) which is documented and freely available for download under the GNU general public license (http://neuroimage.usc.edu/brainstorm). Cortical current maps were computed from the EEG time series using a standardized low-resolution brain electromagnetic tomography (sLORETA), separately for each condition (aware-attended aware-unattended, unaware-attended, unaware-unattended,) and for each subject. Cortical currents were then averaged across subjects and over three time windows of interest (0–50, 150–200, 750–800 ms). Awareness-related sources were assessed by *t*-tests comparing aware (averaged from aware-attended and aware-unattended conditions) and unaware conditions (averaged from unaware-attended and unaware-unattended conditions). Attention-related sources were assessed using the same procedure. Active sources were defined as those containing at least 20 adjacent vertices whose *t*-value exceeded 1.75, corresponding to a *p*-value of 0.05 (uncorrected for multiple comparisons).

## Results

### Behavior

#### Subjective measure of awareness

Subjects gave an “aware” response (presence of a speed-change) in approximately 50% of trials. To confirm that participants did not respond randomly, subjective responses in speed-change-present and speed-change-absent trials were compared. Subjects reported the presence of a speed-change significantly more often (paired *t*-test, *p* < 10^−7^) when the speed-change was present (detection rate, mean ± SEM = 54.9 ± 3.6%) than when it was absent (false-alarm rate, mean ± SEM = 11.7 ± 3.0%). In addition, participants identified the type of the speed-change (accelerated/decelerated) significantly more reliably (paired *t*-test, *p* < 10^−11^) than the speed-change reported as unaware (aware stimuli: mean ± SEM = 94.8 ± 1.1% correct response at the speed-change discrimination task, unaware stimuli: mean ± SEM = 52.5 ± 1.8%). These data indicate that participants were reporting their subjective perception of the presence or absence of a speed-change and not responding randomly. Sorting the behavioral data based on the subjective reports likely corresponds to two objectively distinct cognitive states: an aware state in which subjects were accurate at reporting the speed-change of the stimulus, and an unaware state in which subjects did not report speed-change. We therefore focused our analysis on trials in which subjects reported speed-change and discriminated correctly (aware trials) and those trials in which subjects did not report the speed change and answered randomly on the speed-change discrimination task (unaware trials).

### Attention effect

Attentional benefit is generally associated with faster reaction time(Prinzmetal et al., 2005). In the present study, attentional benefit was expected to lead to a faster response to the speed-change discrimination task for stimuli of the attended color. To minimize the impact of reaction time outliers, trials with reaction times <200 or >1,500 ms were discarded. In addition, a trimming procedure was applied to discard outliers falling outside two SDs around the mean.

A 2(attended, unattended) × 2(aware, unaware) repeated measures ANOVA was conducted on speed-change discrimination rate and reaction time. Reaction time in the attended condition was significantly faster than in the unattended condition [attended, mean ± SEM = 682.47 ± 7.34 ms; unattended, mean ± SEM = 691.97 ± 7.49 ms, *F*_(1, 15)_ = 4.48, *p* = 0.05, $\eta_{\text{p}}^{2}$ = 0.23] Attention had no effect on the discrimination rate of speed-change, [attended, mean ± SEM = 74.2 ± 0.3%; unattended, mean ± SEM = 73.0 ± 0.3%, *F*_(1, 15)_ = 1.66, *p* = 0.22]. A paired *t*-test comparing the detection rate of the subtle speed-change between the attended and unattended conditions revealed no significance difference (attended, mean ± SEM = 55.1 ± 3.5%; unattended, mean ± SEM = 54.6 ± 3.8%; two-tailed paired *t*-test, *p* = 0.56).

### Electrophysiological correlates for color-based attention and awareness

**Figure 3:**
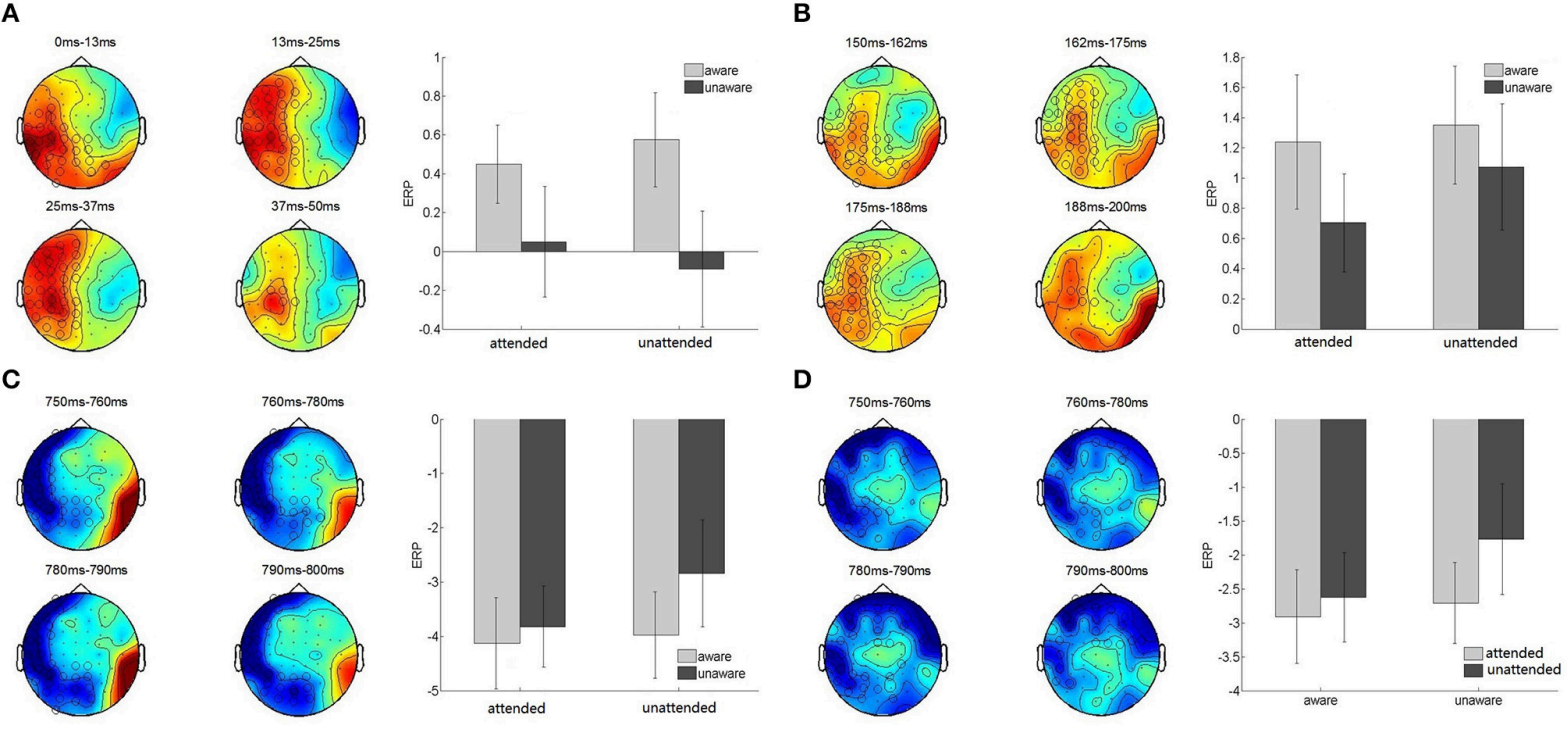
Attention and awareness related clusters. **(A–C)** Awareness-related effects in the 0–50, 150–200, and 750–800 ms post-cue presentation time period. Left, topographical distribution of the significant awareness-related clusters. The color map corresponds to the amplitude of the difference between aware and unaware trials, averaged over the corresponding time-window. Sensors activated in the significant awareness-related clusters are indicated by open circles. Diameters of the open circles are proportional to the duration of the activation of the sensor in the cluster. Right, bar graphs of the averaged ERP activation in the significant positive **(A,B)** and negative **(C)** clusters in the four experimental conditions. Error bars indicate standard error of the mean. **(D)** Attention-related effect in the 750–800 ms post-cue presentation time period. Left, topographical map of the difference between attended and unattended conditions, averaged over the corresponding time-window. Right, bar graph of the averaged ERP activation in the significant negative cluster in the four experimental conditions.

The cluster-based permutation test revealed a significant difference between the aware and unaware conditions in the 0–50 ms post-cue period (*p* < 0.05). There was one positive and three negative clusters of (sensor, time)-samples with a significant difference between conditions in the positive cluster (Monte Carlo *p*-value less <0.025). The averaged ERP activation in the significant positive cluster was computed for each subject and condition, and a 2 (attended, unattended) × 2 (aware, unaware) repeated-measures ANOVA was run. The awareness-related effect was significant [*F*_(1, 15)_ = 8.01, *p* = 0.013, $\eta_{\text{p}}^{2}$ = 0.35] while there was no main effect of attention [*F*_(1, 15)_ = 0.002, *p* = 0.97] or interaction between attention and awareness [*F*_(1, 15)_ = 0.88, *p* = 0.36] (Figure 3A).

The cluster-based permutation test revealed a significant difference between the aware and unaware conditions (*p* < 0.05) in the 150–200 ms post-cue period. There was one positive and one negative cluster of (sensor, time)-samples with a significant difference between conditions in the positive cluster (Monte Carlo *p* < 0.025). The averaged ERP activation in the significant positive cluster was computed for each subject and condition. A 2 (attended, unattended) × 2 (aware, unaware) repeated-measures ANOVA was run and revealed an awareness-related effect [*F*_(1, 15)_ = 13.48, *p* < 0.01, $\eta_{\text{p}}^{2}=$ 0.47]. However, there was no main effect of attention [*F*_(1, 15)_ = 1.46, *p* = 0.25] or interaction between attention and awareness [*F*_(1, 15)_ = 0.41, *p* = 0.53] (Figure 3B).

The cluster-based permutation test revealed a significant difference between the aware and unaware conditions (*p* < 0.05). There was one significant negative cluster of (sensor, time)-samples (Monte Carlo *p* < 0.025). The averaged ERP activation in the significant negative cluster was computed for each subject and condition. A 2 (attended, unattended) × 2 (aware, unaware) repeated-measures ANOVA revealed a main effect of awareness [*F*_(1, 15)_ = 8.87, *p* = 0.01, $\eta_{\text{p}}^{2}=$ 0.37). In addition there was a main effect of attention [*F*_(1, 15)_ = 4.78, *p* = 0.045]. There was no interaction between awareness and attention [*F*_(1, 15)_ = 2.17, *p* = 0.16] (Figure 3C).

A cluster-based permutation test revealed a significant difference between the attended and unattended conditions (*p* < 0.05) in the 750–800 ms post-cue period. Two negative clusters of (sensor, time)-samples were revealed with only one significant cluster (Monte Carlo *p* < 0.025). The averaged ERP activation in the significant negative cluster was computed for each subject and condition and a repeated measures ANOVA revealed a significant attention-related effect [*F*_(1, 15)_ = 6.56, *p* = 0.02, $\eta_{\text{p}}^{2}=$ 0.30] and a main effect of awareness[*F*_(1, 15)_ = 6.56, *p* = 0.02]. There was no interaction between awareness and attention [*F*_(1, 15)_ = 1.60, *p* = 0.23] (Figure 3D). The differences between the attended and unattended conditions were largely observed at 750–800 ms post-cue, while differences in awareness were observed earlier and were more sustained in the following three time-widows: 0–50, 150–200, and 750–800 ms. The attention and awareness-related effects therefore exhibit distinct time-courses. Interestingly, there was a difference between the aware and unaware trials prior to the onset of the target, in the 0–50 ms time-widow.

### Source localization

**Figure 4:**
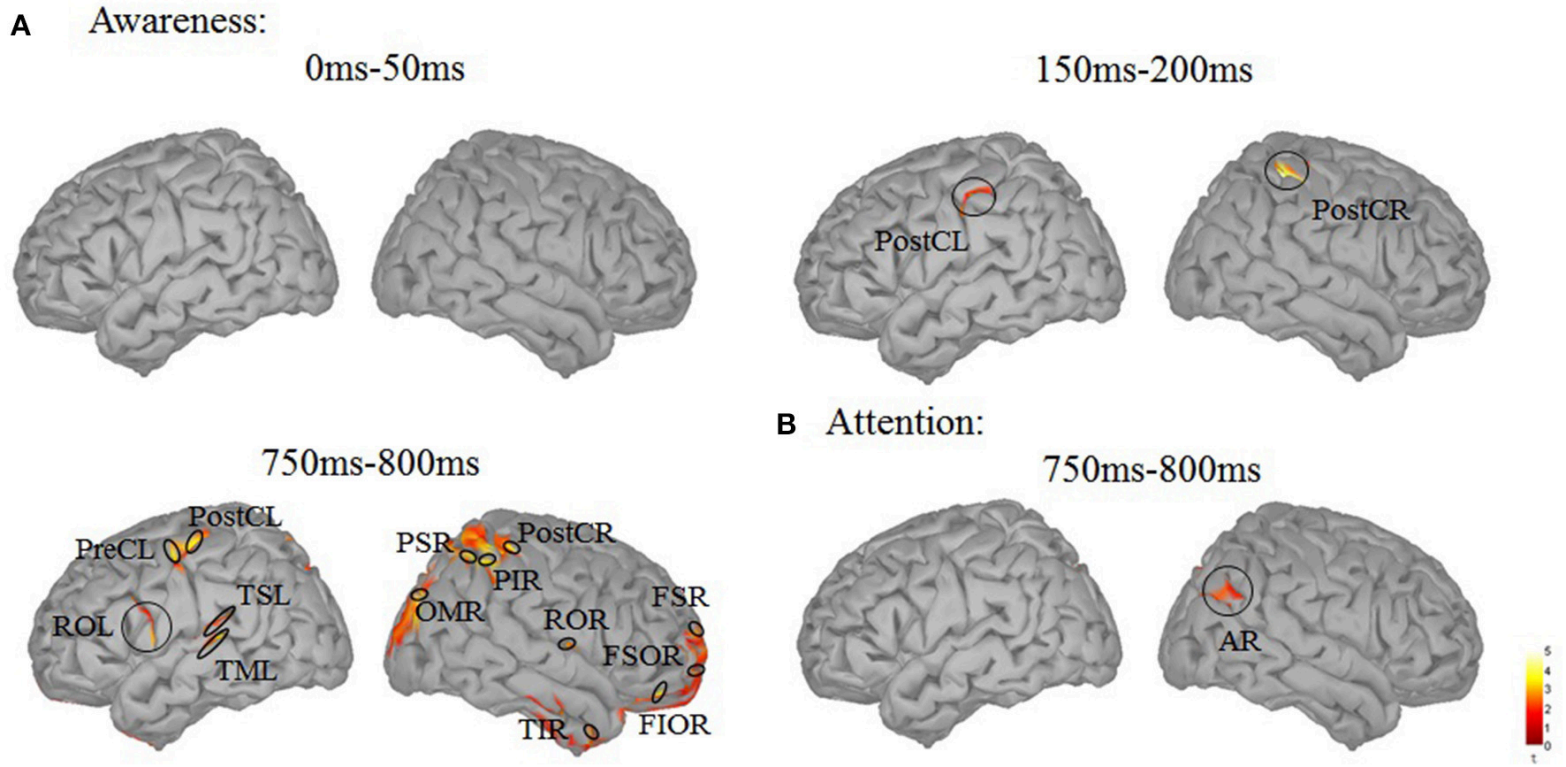
Source localization of the main effects. **(A)** Contrast between the aware and unaware conditions, in the 0–50, 150–200, and 750–800 ms time-windows. Significant sources were observed in the bilateral central cortex (150–200 ms) and in widely distributed areas [including bilateral postcentral gyrus (PostC), left precentral gyrus (preCL), bilateral Rolandic operculum (OR), left superior temporal lobe (TSL), left middle temporal lobe (TML), right orbital inferior frontal lobe (FIOR), right superior frontal lobe (FSR), right orbital superior frontal lobe (FSOR), right middle occipital lobe (OMR), right inferior parietal lobe (PIR), right superior parietal lobe (PSR) and right inferior temporal cortex (TIR)]. **(B)** Contrast between attended and unattended trials, in the 750–800 ms time-window. One significant source was observed in the right angular gyrus (AR).

To uncover brain areas contributing to these two main effects, a distributed source model was used. We modeled neural responses to aware and unaware stimuli separately and computed the difference in the three time windows (0–50, 150–200, 750–800 ms). In the 150–200 ms time window, significant differences between seen and unseen trials were localized bilaterally in the post-central cortex (Figure 4A). The MNI coordinates of the two areas where there was awareness-related activation are presented in Table 1. In the 750–800 ms time window, a significant difference was observed over a wide area (including bilateral post-central gyrus, left precentral gyrus, bilateral Rolandic operculum, left superior temporal lobe, left middle temporal lobe, right orbital inferior frontal lobe, right superior frontal lobe, right orbital superior frontal lobe, right middle occipital lobe, right inferior parietal lobe, right superior parietal lobe, and right inferior temporal cortex, Figure 4A). In the 750–800 ms time window, a significant difference in color-congruency was localized to the right angular gyrus, as depicted in Figure 4B.

**Table 1:** MNI coordinates (mm) of the activated regions.

	**Times**	**Regions**		**Coordinates(mm)**		
				***x***	***y***	***z***
Awareness	0–50 ms	OIL	Occipital_Inf_L	−18.5	−100.8	−8.3
	150–200 ms	FL	Fusiform_L	−32.6	−58.9	−16.5
		PostCL	Postcentral_L	−48	−17.3	39.7
		PostCR	Postcentral_R	28	−42.8	60.5
		OIR	Occipital_Inf_R	21.2	−101.6	−9.8
	750–800 ms	PostCL	Postcentral_L	−42.9	−17.8	54.8
		PreCL	Precentral_L	−41.7	−7.9	54.9
		ROL	Rolandic_Oper_L	−59.3	2	7.5
		TSL	Temporal_Sup_L	−55.3	−38.1	20.4
		TML	Temporal_Mid_L	−59.4	−32.3	9
		FIOR	Frontal_Inf_Orb_R	42.1	45.8	−19
		FSR	Frontal_Sup_R	22.5	66.7	15.7
		FSOR	Frontal_Sup_Orb_R	22.3	65.7	−6.5
		OMR	Occipital_Mid_R	29.1	−84.3	37.4
		PIR	Parietal_Inf_R	46.6	−51.6	56
		PSR	Parietal_Sup_R	37.1	−55.8	59.6
		PostCR	Postcentral_R	45.3	−35.4	63.2
		ROR	Rolandic_Oper_R	52.6	−3.3	9.3
		TIR	Temporal_Inf_R	48	6.6	−46.4
Attention	750–800 ms	AR	Angular_R	50.2	−68.4	36.7

**Figure 5:**
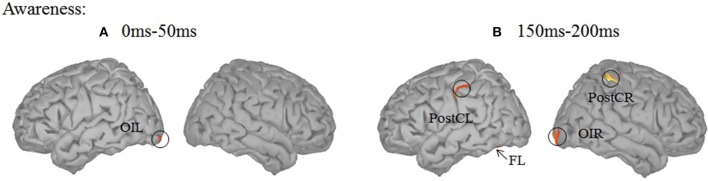
Source localization using a more liberal threshold. **(A)** Contrast between the aware and unaware conditions in the 0–50 ms time-window. A significant source was observed in the left inferior occipital cortex (OIL). **(B)** Contrast between the aware and unaware conditions in the 150–200 ms time-window. Significant sources were observed in the bilateral post-central gyrus (PostC), left inferior occipital cortex (OIL) and left fusiform gyrus (FL).

In the analysis presented above, a region was considered differentially activated if it contained at least 20 adjacent vertices (out of 15,000) with an individual *p*-value smaller than 0.05. With this threshold, awareness-related effects were absent in the 0–50 ms time window and were confined to motion-related regions in the 150–200 ms time window. The early activation in the visual cortex may be confined in space. Therefore, by using a more liberal threshold (9 adjacent vertices with *p* < 0.05), awareness-related activation was revealed in the left inferior occipital cortex for two time ranges (0–50 and 150–200 ms) while activation in the left fusiform cortex was found in the 150–200 ms time window only (Figure 5).

## Discussion

The current study aimed to elucidate the nature of the relationship between the neural mechanisms of feature-based exogenous attention and those of visual awareness. Exogenous attention to color and visual awareness were manipulated simultaneously but independently within a single paradigm, using physically identical stimuli. Behaviorally, color-based exogenous attention decreased response-time during the speed-change discrimination task. Activity in clusters of sensors (based on time and location) varied according to either color-based exogenous attention or awareness and identified the existence of neural correlates of feature-based exogenous attention and visual awareness. There was greater negative activation in response to attended stimuli observed mostly within a latency of 750–800 ms post-cue. Conversely, neural correlates of awareness were identified in three time-widows: 0–50, 150–200, and 750–800 ms. The awareness-related effect was exhibited even prior to the onset of the target. The results therefore suggest that exogenous attention and awareness-related effects exhibit distinct neural time-courses.

### Awareness-related effects

Using stimuli that were physically identical, we observed awareness-related neural responses at three time periods (0–50, 150–200, and 750–800 ms). These time periods are consistent with those identified in previous studies on visual awareness (Pins, 2003; Roeber et al., 2008). The awareness-related differences in the 150–200 ms time period may reflect an early step in the emergence of consciousness (Koivisto and Silvanto, 2012). The contribution of brain activity during this period may be critical for feature detection. The source localization results presented here are in agreement with previous EEG studies linking the inferior occipital and fusiform gyri (Vanni et al., 1996; Koivisto et al., 2010; Liu et al., 2012) and post-central gyrus activity (Planetta and Servos, 2012) with visual awareness. The recurrent extrastriate-V1 activity, which has been proposed to be a necessary component of consciousness (Pascual-Leone and Walsh, 2001; Silvanto et al., 2005), is somewhat absent in the current study compare between conscious and unconscious condition. It is of-cause caused by the lack of sensitivity of our current manipulation. It also could due to the fact that this recurrent activity is necessary for both aware and unaware perception (Koivisto et al., 2010). Later awareness-related differences observed in the 750–800 ms time window may reflected a distributed awareness-related activation, similar to that seen in other studies (after 300–400; Del Cul et al., 2007; Lamy et al., 2009; Railo and Koivisto, 2009; Melloni et al., 2011). The activation included some typical visual areas and frontal-parietal networks, often reported to be correlated with awareness-related activity. Some of the frontal cortex activity, such as the right orbital inferior and superior frontal cortex may have arisen from eye-movement. Future studies should control for this possibility by using an eye tracker. The current data therefore, support both an early and late awareness-related neural response. Interestingly, there was an awareness-related effect even prior to the onset of the target in the first time period (0–50 ms). These results are in agreement with previous studies showing predictive neural activity and may reflect a less-reliable effect mediating visual consciousness (Haynes and Rees, 2005; Wyart and Tallon-Baudry, 2009; O'Shea et al., 2013). Inferior occipital activity mediated visual consciousness in this time range.

The neural correlates of visual awareness were distinct from color-based exogenous attention, thereby extending previous results obtained from research on endogenous attention (Koivisto et al., 2006; Koivisto and Revonsuo, 2008; Wyart and Tallon-Baudry, 2008). While the connection between attention and consciousness is stronger when attention is under exogenous, as opposed to endogenous control (Chica et al., 2010, 2012), exogenous attention and visual consciousness are separable under certain circumstances. From a neural view-point, it seems that visual awareness and exogenous attention are independent processes, similar to endogenous attention.

### Attention-related effects

Most often, the neural correlates of color-based attention have been reported in the 150–300 ms interval (Harter et al., 1982; Hillyard and Münte, 1984; Anllo-Vento et al., 1998; Stelt et al., 1998), although earlier attentional modulations have been reported when distractors compete with the target (Zhang and Luck, 2008). In the current study, no significant attention-related modulation was identified in these time windows, contrary to what would be expected from the literature (Corbetta et al., 1991; Motter, 1994; Anllo-Vento et al., 1998; Müller et al., 2006). It may be that our study lacked sensitivity. Alternatively, the attentional mechanisms recruited in the present experiment may affect decisional processes, while perceptual processes were generally unaffected. The pattern of behavioral results, which consists of a decrease in reaction times in attended trials, without a concomitant increase in performance, may indicate such an attentional influence at the decisional level (Prinzmetal et al., 2005). In addition, our results confirm previous EEG studies that link angular gyrus activity with feature-based attention and visual feature binding (Koivisto and Silvanto, 2012).

## Conclusions

The current study identified neural correlates of visual awareness that are distinct from color-based exogenous attention and therefore extend the previously observed distinction between neural correlates of endogenous attention and visual awareness (Koivisto et al., 2006; Wyart and Tallon-Baudry, 2008; Davoodi et al., 2015) to exogenous attention. The results suggest that color-based attention is at least partly distinct from awareness at the neural level. The neural correlates of awareness were identified in several time periods. There were early differences after the onset of the target; later, more broadly distributed differences and even slight differences prior to the onset of the target, which may reflect a less-reliable activation mediating visual consciousness.

## Author contributions

YL designed experiments; YC, XW, YY, and YL conducted experiments, analyzed data; YL wrote the paper.

### Conflict of interest statement

The authors declare that the research was conducted in the absence of any commercial or financial relationships that could be construed as a potential conflict of interest.
